# Changes in Functional Properties and In Vitro Digestibility of Black Tartary Buckwheat Starch by Autoclaving Combination with Pullulanase Treatment

**DOI:** 10.3390/foods13244114

**Published:** 2024-12-19

**Authors:** Faying Zheng, Fuxin Nie, Ye Qiu, Yage Xing, Qinglian Xu, Jianxiong Chen, Ping Zhang, Hong Liu

**Affiliations:** 1 School of Food and Health, Beijing Technology and Business University, Beijing 100080, China; zfyasdzxc2021@163.com; 2 Key Laboratory of Grain and Oil Processing and Food Safety of Sichuan Province, College of Food and Bioengineering, Xihua University, Chengdu 610039, China; niefuxin1008@163.com (F.N.); qiuye57995@163.com (Y.Q.); xuqinglian01@163.com (Q.X.); gyliuhong@126.com (H.L.); 3 Huantai Biotechnology Co., Ltd., Chengdu 610225, China; cjx81607078@aliyun.com (J.C.); htzp2019@163.com (P.Z.)

**Keywords:** black Tartary buckwheat starch, physicochemical properties, rheological properties, in vitro digestibility properties, pullulanase

## Abstract

The processing properties of resistant starch (RS) and its digestion remain unclear, despite the widespread use of autoclaving combined with debranching in its preparation. In this study, the physicochemical, rheological and digestibility properties of autoclaving modified starch (ACB), autoclaving–pullulanase modified starch (ACPB) and native black Tartary buckwheat starch (NB) were compared and investigated. The molecular weight and polydispersity index of modified starch was in the range of 0.15 × 10^4^~1.90 × 10^4^ KDa and 1.88~2.82, respectively. In addition, the SEM results showed that both modifications influenced the morphological characteristics of the NB particles, and their particles tended to be larger in size. Autoclaving and its combination with pullulanase significantly increased the short-range ordered degree, resistant starch yield and water- and oil-absorption capacities, and decreased the syneresis properties with repeated freezing/thawing cycles. Moreover, rheological analysis showed that both ACB and ACPB exhibited shear-thinning behavior and lower gel elasticity as revealed by the power law model and steady-state scan. The degradation of starch chains weakened the interaction of starch molecular chains and thus changed the gel network structure. The in vitro digestion experiments demonstrated that ACB and ACPB exhibited greater resistance to enzymatic digestion compared to the control, NB. Notably, the addition of pullulanase inhibited the hydrolysis of the ACB samples, and ACPB showed greater resistance against enzymatic hydrolysis. This study reveals the effects of autoclaving combined with debranching on the processing properties and functional characteristics of black Tartary buckwheat starch.

## 1. Introduction

Tartary buckwheat (TB) is a pseudocereal plant cultivated in harsh environments. Studies have reported that TB may help prevent hyperglycemia, hyperlipidemia, diabetes, obesity and related inflammatory conditions [1,2,3,4]. Furthermore, TB seeds consist of more than 70% starch (dry weight) and are therefore a potential new source of starch [5,6]. The properties and functions of Tartary buckwheat starch (TBS) have attracted intensive research attention [7,8,9,10]. For example, after extrusion treatment, the water absorption index and resistant starch content of TB flour increased significantly, and more slowly digested starch in the native TB flour was obtained by improved extrusion processing techniques [11]. Liu et al. reported that annealing-treated TBS exhibited a significant increase in amylose content and water absorption, while its solubility and oil absorption gradually decreased [12]. The quercetin may limit the swelling, gelatinization and retrogradation of TBS, and in particular, the addition of Ca(OH)_2_ to the TBS and quercetin mixed system may further alleviate the retrogradation of starch and improve the gel strength [13]. Black Tartary buckwheat is a variety of TB, often referred to as the “Black Pearl” due to its dark hull and abundant nutritional value [14,15]. However, there has been very little research on the functional properties of black Tartary buckwheat starch.

Starch is a natural polymer compound formed by the polymerization of several glucose units, which is an important source of energy in the daily diet. Digestibility is the primary physiological characteristic of starch. Based on differences in digestive properties, starch can be classified as rapidly digestible starch (RDS), slowly digestible starch (SDS) and resistant starch (RS) [16]. With the rising prevalence of diabetes, starch with lower digestibility has become a growing concern for consumers [17,18]. The addition of appropriate amounts of resistant starch to food ingredients can enhance the textural properties and flavor of the product. For example, Punia et al. showed that the incorporation of RS into noodles resulted in reduced chewiness and maintained an acceptable overall quality of the noodles [19]. A significant improvement in cookie quality was observed with the addition of 50% cross-linked wheat RS [20]. In general, the production of sushi and porridge requires starch with high viscosity and moderate digestibility, while fried puff pastry production demands starch with low viscosity and low digestibility, and feed production benefits from starch with moderate viscosity and rapid digestion [21,22,23]. Resistant starch with low water holding capacity may improve the softness of dough and can also be incorporated into jams to stabilize the structure and maintain quality [20,24]. Therefore, in order to meet those requirements, native starch is subjected to different modifications to obtain new properties such as resistance to digestion, increased viscosity, and enhanced moisture retention to make it suitable for various applications.

Autoclaving and debranching are widely employed starch modification techniques that effectively increase resistant starch content [25]. Compared to chemical modification, these processes do not cause environmental pollution and are more acceptable to consumers. Autoclaving usually leads to starch gelatinization and destruction of the original crystal structure. The starch paste is cooled at low temperatures, resulting in the reorganization of new crystals [26]. The addition of pullulanase to gelatinized starch is a common method for producing linear dextran chains. Due to the strong mobility and molecular orientation of short linear chains, the debranched starch might rapidly form a double helical aggregate during reorganization [27]. Studies indicated that the debranching and recrystallization treatments significantly improved the digestion resistibility [28,29], solubility index and swelling index of native starch [30]. These results suggest that the reorientation and rearrangement of starch molecules may affect the structure of starch and thus its functional properties.

The present research could give a better understanding of the effect of autoclaving combined with debranching on black Tartary buckwheat starch, because the combined modification would change the molecular structure, granular structure, rheological behavior, hydration properties and digestive properties of the starch. In addition, such properties would greatly affect the final quality of starch-based foodstuffs. Previous studies have shown that autoclaving and pullulanase treatments could significantly increase the resistant starch content in black Tartary buckwheat [31]. Therefore, a comparison of the differences in physicochemical, rheological and digestive properties of BTB starch between autoclaving and pullulanase was examined in this study. The results may contribute to the development of products with the desired function of black Tartary buckwheat starch.

## 2. Materials and Methods

### 2.1. Main Material

Black Tartary buckwheat whole flour was obtained from Huantai Biotechnology Co., Ltd. (Xichang, China). Pullulanase (1000 ASPU/mL), amyloglucosidase (100,000 U/g) and α-amylase (20,000 U/g) were purchased from Source Leaf Chemical Co., Ltd. (Shanghai, China). The chemicals used were of analytical grade. The amylose starch kit (BC4260) was purchased from Solabio Technology Co., Ltd. (Beijing, China) and the detection method used was visible spectrophotometry. The composition in terms of starch, moisture, protein, fat and flavonoids in black Tartary buckwheat native starch was 89.95%, 9.08%, 0.20%, 0.34% and 0.78 mg/kg, respectively.

### 2.2. Isolation of Black Tartary Buckwheat Starch

The starch was isolated from black Tartary buckwheat whole flour using the method of Liu et al. [32]. Briefly, the ground whole black buckwheat powder was mixed with sodium hydroxide solution (0.3%, *w*/*v*) in a ratio of 1:10 and stirred continuously for 20 min, then left for 24 h. After slowly decanting the supernatant, the precipitate was washed with distilled water until a compact white starch layer was obtained. Subsequently, the resulting white sediment was collected, dried in an oven at 55 °C for 10 h, ground and passed through a 100-mesh sieve. The product obtained was named black Tartary buckwheat native starch (NB).

### 2.3. Preparation of Modified Black Tartary Buckwheat Starch

The debranched starch was prepared using the method described by Zhou et al. [33]. The configured suspension (5%, *w*/*v*) was placed in an autoclave for 20 min (121 °C) to completely gelatinize the starch. Then, the starch paste was treated with pullulanase (14 ASPU/g starch) at 50 °C for 4.43 h. These parameters were chosen before the experiment. The enzyme was inactivated by boiling it in a water bath. Next, the starch paste was placed in a refrigerator at 4 °C for 24 h, subsequently freeze-dried (48 h) and sieved to obtain ACPB samples. In the modification treatment, NB without debranching was labeled as ACB samples.

### 2.4. Starch Granule Morphology, Size Distribution and Color

Scanning electron microscopy (SEM, Thermo Fisher, Waltham, MA, USA) was used to observe the morphology of starches. The images were obtained at an accelerating voltage of 7.00 kV. For native and modified starches, photographs were taken at magnifications of 500, 1000 and 2000 times, respectively. Starch granules were coated with gold under vacuum before measurement. A laser-light scattering particle size analyzer was used to record the size distribution of black Tartary buckwheat starch and modified starch following the method reported previously [34]. The color of the samples was determined using the colorimeter (WF32, Weifu Optoelectronics Technology Co., Shenzhen, China), as described by Ashwar et al. [35]. The equipment was standardized by a calibration plate before each measurement. Three different positions were selected for measurement on each starch sample to obtain uniform color measurements, and L*, a* and b* values were recorded.

### 2.5. Measurement of Molecular Weight of Starch

The molecular weight of starch was determined according to the method from previous research [36], and each sample was mixed with DMSO (5 mL) and heated in a water bath at 80 °C for 3 h. The Ohpak SB-804 HQ (300 × 8 mm) column was used and kept at 60 °C. The flow rate of the mobile phase was 0.3 mL/min. The filtrate (0.2 mL) was injected into a U3000 system consisting of an Optilab T-rEX detector (Wyatt technology, Goleta, CA, USA) and a DAWN HELEOS II detector (Wyatt technology, Goleta, CA, USA). The electronic output of the collection detector was used for calculating the molecular weight of the sample according to the Mark–Houwink Equation, and the obtained data were analyzed by Astra version 6.1 software.

### 2.6. Short-Range Ordered Structure

According to the method of Espinosa-Solis et al. [37], using Fourier transform infrared spectroscopy (FTIR, FTIR-1500, Nicolet, WI, USA), spectra of all samples were obtained using an FTIR spectrometer equipped with an ATR (attenuated total reflection) accessory. The samples were uniformly coated on the surface of the ATR crystals. The scanning range was 600 to 4000 cm^−1^, and the data were recorded and then analyzed for the short-range ordered structure of starch.

### 2.7. Water Solubility (WS), Swelling Power (SP), Water Absorbing Capacity (WAC) and Oil Absorbing Capacity (OAC)

The WS and SP index of starch samples were determined using the method of Xu et al. [31] with slight modifications. In total, 0.3 g of starch was mixed with 15 mL of distilled water in a centrifuge tube and subsequently heated at 50 °C, 60 °C, 70 °C, 80 °C and 90 °C for 30 min, respectively. Samples were centrifuged at 4000 rpm for 20 min, the supernatant was collected and the precipitate was weighed. The collected supernatant was transferred to an aluminum box, dried in an oven at 105 °C and then weighed again. Furthermore, the OAC and WAC index of starch samples were determined with the earlier method [35]. Then, 100 mg of starch was added to a centrifuge tube containing 10 mL of corn oil and mixed every 5 min for a total of 30 min. Next, the starch samples were centrifuged at 4000 rpm for 20 min, and the upper liquid layer was slowly decanted and the weight of the precipitate was recorded.

$$\mathrm{WS}\;(\%)=\frac{\mathrm{weight}\;\mathrm{of}\;\mathrm{the}\;\mathrm{dried}\;\mathrm{supernatant}}{\mathrm{weight}\;\mathrm{of}\;\mathrm{dry}\;\mathrm{starch}}\;\times \;100$$

$$\mathrm{SP}\;(\%)=\frac{\mathrm{weight}\;\mathrm{of}\;\mathrm{the}\;\mathrm{sediment}}{\mathrm{weight}\;\mathrm{of}\;\mathrm{dry}\;\mathrm{starch}(1-\mathrm{WS}/100)}\;\times \;100$$

$$WAC\;\mathrm{or}\;OAC\;(g/g)=\frac{\mathrm{weight}\;\mathrm{of}\;\mathrm{wet}\;\mathrm{starch}-\mathrm{weight}\;\mathrm{of}\;\mathrm{dry}\;\mathrm{starch}}{\mathrm{weight}\;\mathrm{of}\;\mathrm{dry}\;\mathrm{starch}}\;\times \;100$$

### 2.8. Freeze–Thaw Stability and Light Transmittance

The freeze–thaw stability of starch was estimated using the method reported elsewhere [35]. First, the starch suspension was stirred continuously in a water bath for 30 min at 80 °C. The cooled starch paste was refrigerated at −18 °C for 22 h and then thawed in a water bath at 25 °C for 2 h. Refrigeration and thawing were proceeded for 5 cycles. Next, the starch was centrifuged with 5000 rpm for 20 min at 10 °C, and then the released water was measured. Moreover, the transmittance of starch was determined according to the previous method [38]; the starch samples were mixed with deionized water (6%, *w*/*v*) and gelatinized in a boiling water bath for 30 min. The starch paste was cooled to 25 °C and placed in a refrigerator at 4 °C for 24 h. Then, the absorbance values were measured by UV spectrophotometer (UV2400, Sunny Hengping Scientific Instruments, Shanghai, China) at 640 nm every 24 h, with deionized water as the control.

### 2.9. Rheological Measurement

#### 2.9.1. Steady Shear

The steady shear of starch paste was estimated using a rheometer (MCR-301, Anton Paar, Austria) based a previous method [39]. First, starch and deionized water were mixed (6%, *w*/*v*) and heated in a water bath at 95 °C for 30 min, with stirring performed every five minutes. Next, the starch paste was left to cool at room temperature for 1 h. Then, the sample was loaded onto a rheometer plate (25 mm diameter) at 25 °C, and the shear rate (γ) was increased from 0.01 to 300 s^−1^. The apparent viscosity (η) and shear stress (τ) were specified as dependent variables of the shear rate. Moreover, the obtained data were fitted to a power law model with the following equation:

$$\tau =k\gamma^{n}$$

In the above function, K represents the consistency coefficient, n refers to the fluidity behavior coefficient, n > 1 means fluid with shear thickening behavior, n < 1 means fluid with shear thinning behavior and n = 1 means Newtonian fluids

#### 2.9.2. Dynamic Viscoelasticity

The frequency scan was performed following the previous method with some modifications [40]. The linear viscoelastic region of all starch pastes was identified prior to testing. The measurements were performed at 25 °C with a fixed strain at 1% and an angular frequency range from 1 loaded to 10. The modulus of elasticity (G′), modulus of loss (G″) and loss coefficient (tanδ) were considered dependent variables of the angular frequency.

### 2.10. In Vitro Digestibility

The in vitro digestibility of native and modified starches was performed using the method of Englyst et al. [41], modified by Zhang et al. [42]. The starch and acetate buffer were mixed in centrifuge tubes, mixed well with a vortex mixer and placed at 37 °C for 15 min. Then, the enzyme mixture solution (amyloglucosidase 15 U/mL and α-amylase 290 U/mL) was added to the above-mentioned centrifuge tubes and incubated at 37 °C. The tubes were removed at different time intervals (0, 20, 60, 90, 120, 180, 210 and 240 min), and ethanol was added to inactivate the enzyme. The mixture was centrifuged, the supernatant was taken and the glucose content was determined by the 3,5-dinitrosalicylic acid (DNS) method after the corresponding time. In addition, RDS, SDS and RS were calculated by the following.

$$\mathrm{RDS}(\%)=G_{20}\times 0.9\times 100$$

$$\mathrm{SDS}(\%)=(G_{120}-G_{20})\times 0.9\times 100$$

RS(%)=TS−RDS−SDS

In the above equation, G_20_ and G_120_ refer to the glucose content of hydrolysis within 20 and 120 min, respectively, and TS is the total starch content of the sample.

The data measured above were fitted by a first-order equation for the kinetics of the starch reaction under in vitro digestion enzymatic digestion, with the following equation.

$$C=C\text{∞}(1-e^{-\mathrm{kt}})$$

where C (%) is the total amount of starch digested at digestion time t; C∞(%) is the total amount of starch digested at 240 min; and k (min^−1^) is the rate coefficient. The starch digestion was determined using a method reported elsewhere [42].

### 2.11. Statistical Analysis

The chain length distribution experiment was conducted once, and other measurements were repeated at least three times and expressed as mean ± standard deviation. Data were analyzed for ANOVA using SPSS 22.0 software, and significant differences between data were determined using Duncan multiple comparisons. Data were plotted using origin 2021 and GraphPad 8.0 software.

## 3. Results and Discussion

### 3.1. Apparent Amylose Content

The apparent amylose content (AAC) in NB after various modification treatments was determined using an amylose kit. The AAC of NB, ACB and ACPB are shown in Table 1. The AAC in ACB samples significantly increased from 12.95% to 16.28% following autoclaving treatment. Furthermore, ACPB exhibited the greatest elevation of AAC (from 12.95% to 24.15%) and significant (*p* < 0.05) difference between autoclaving and autoclave-debranching modifications.

Meanwhile, the AAC of NB (12.95%) was lower than the amylose content reported previously in Tartary buckwheat (29.1%) and common buckwheat starch (17.1%) [32,43]. In addition, the starch from buckwheat harvested in different seasons may have similar AAC value. Differences in the AAC value of different varieties of buckwheat may be attributed to the biological origin, genetics and environment [31]. The results showed that the AAC of the samples was affected by the modification treatments. For ACB, there was a numerical increase of 3.33 percentage points in AAC value, suggesting that the autoclave treatment may promote the degradation of starch long chains. Sun et al. [27] studied the autoclaved purple sweet potato starch and obtained similar results. However, the highest elevation of AAC value was observed in ACPB samples. This is due to the fact that pullulanase could specifically cleave the α-1,6 glycosidic bond of amylopectin chain, leading to more linear chains. A similar phenomenon of increased AAC value has been observed in debranched modified maize starch and oat starch [44,45]. The chemical composition of native and modified starch from black Tartary buckwheat may depend on the modification and extraction applied. Further investigation should be conducted to evaluate the effects of different extraction methods and modification techniques (physical, chemical, or enzymatic methods) on the quality of black Tartary buckwheat starch.

### 3.2. SEM, Size Distribution and Color

**Figure 1:**
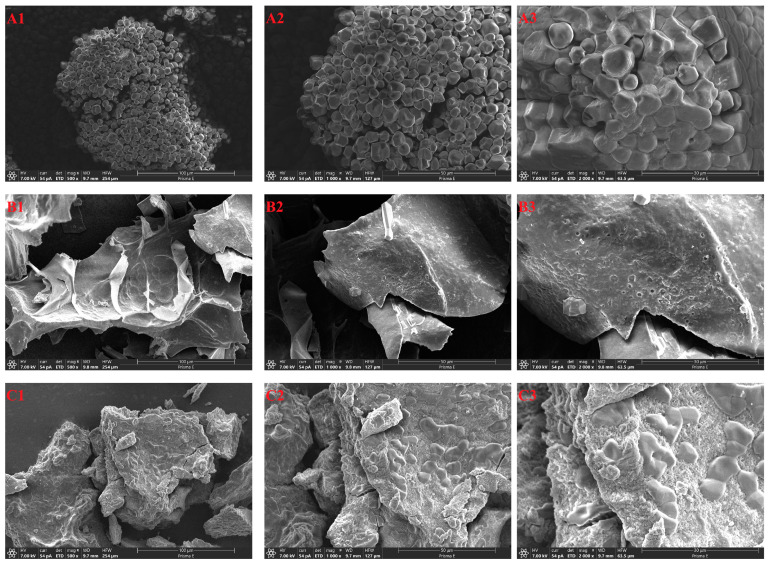
SEM micrographs of NB (**A1**,**A2**,**A3**), ACB (**B1**,**B2**,**B3**) and ACPB (**C1**,**C2**,**C3**) (magnification: 500×, 1000× and 2000×). NB, black Tartary buckwheat native starch; ACB, black Tartary buckwheat starch subjected to autoclaving treatment; ACPB, black Tartary buckwheat starch subjected to autoclaving–pullulanase combined treatment.

**Figure 2:**
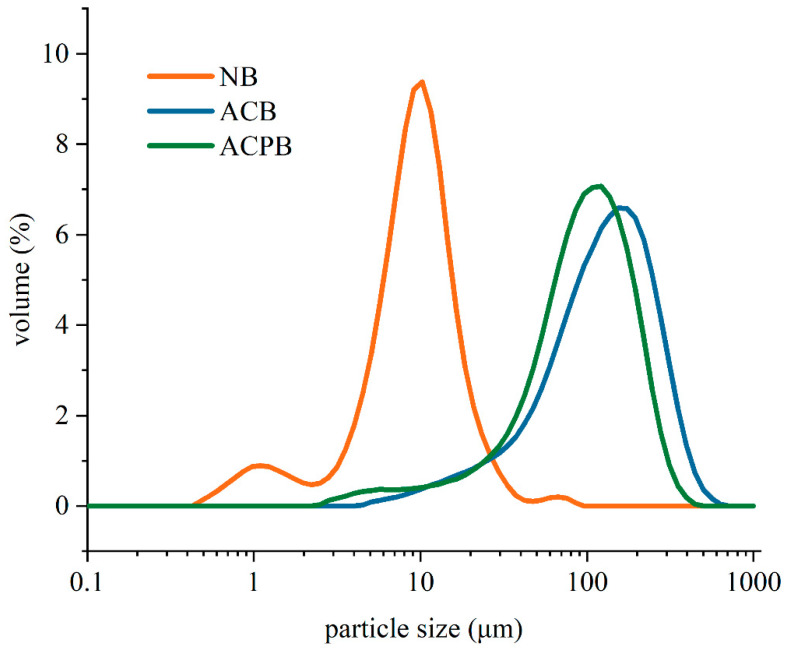
Particle size distribution of NB, ACB and ACPB. NB, black Tartary buckwheat native starch; ACB, black Tartary buckwheat starch subjected to autoclaving treatment; ACPB, black Tartary buckwheat starch subjected to autoclaving–pullulanase combined treatment.

The morphological changes in native and modified starch morphology were investigated by SEM. The NB showed rounded, individual particles with some concavities on smooth surfaces (Figure 1(A1–A3)). Starch morphology is influenced by the cultivation conditions, genetic attributes and processing methods. Fan Zhu [5] showed that the shape of buckwheat starch granules was mostly polygonal, spherical and oval, with a size range between 2 and 19 μm [32,46,47]. After autoclaving or debranching treatment, the original structure of the native starch was destroyed, and the modified starch was reintegrated to show flake (Figure 1(B1)) or amorphous block (Figure 1(C1)) characteristics. After the autoclaving and debranching treatment, the size distribution of starch shifted toward larger granule sizes (Figure 2). Table 1 shows the average particle size distributions of starch granules. The peak particle sizes of NB, ACB and ACPB were 9.57, 155.60 and 105.53 μm, respectively. Additionally, D[4,3] reflects the average diameter of the area, while D[3,2] represents the average diameter of the volume. After autoclave treatment, D[3,2] and D[4,3] of ACB increased significantly (*p* < 0.05) from 5.63 μm to 66.73 μm and 15.12 μm to 136.13 μm. In comparison with ACB, the D[3,2] and D[4,3] decreased in ACPB, from 66.73 μm to 47.12 μm and 136.13 μm to 103.37 μm. This was consistent with the results of SEM. The D[3,2] and D[4,3] of the two modified starches were recorded in the following order: ACB > ACPB > NB. In this study, the color properties of starch were determined. The results showed that NB had a higher L* value (98.99 ± 0.46), lower a* value (−0.74 ± 0.07) and lower b* value (2.42 ± 0.13) than ACB and ACPB samples. In addition, the L* values of ACB and ACPB reduced dramatically, while the a* and b* values of ACB and ACPB increased significantly after autoclaving or autoclaving debranching co-treatments. The color of starch is an important parameter that determines its use in food processing.

In fact, the SEM technique can be used to observe the morphology of modified starch. NB granules were mostly round or polygonal with concave surfaces. The observation was consistent with the results reported by Gao et al. [48]. A significant difference in the shape of the modified starch was observed when compared to the NB sample. The ACB and ACPB samples lost their granule shape and formed a larger, irregular structure. The reason can be attributed to the swelling and rupture of gelatinized starch granules and recrystallization [30]. Due to the modification of debranching, the apparent structures of ACPB and ACB differ. During gelatinization and retrogradation, starch chains escape from the granules and rearrange during further cooling, which may explain the morphological differences in the modified starches [31]. ACPB presented a continuous network structure with a rough surface. This might be attributed to the generation of linear fragments with shorter chain lengths that are more suitable for retrogradation than ACB as a consequence of the application of pullulanase treatment, which eventually formed mixed crystal bundles through hydrogen bonding. Similar results were reported by Zhang and Jin [49]. Resistant starch modified with a combination of α-amylase and pullulanase showed a more compact structure compared to native corn starch. Previous studies have shown that the rice starch formed an irregular continuous network structure after dual autoclaving retrogradation modification, and the chestnut starch showed a spongy-like and cohesive structure after autoclave–pullulanase hydrolysis [35,50]. Moreover, the native starch granules were much smaller than that of recrystallized starch, which was consistent with the results reported by Xu et al. [31]. The morphology of modified starch tended to a larger size, which may contribute to the reduction in enzymatic sensitivity of NB to digestion. Similar results had previously been reported for arrowhead-derived resistant starch [51]. Autoclaving treatment breaks the hydrogen bonds between starch molecules, while pullulanase hydrolyzes the α-1,6 glycosidic bonds in the chains of branched starch molecules, thereby increasing the number of linear chains. Moreover, the change in color was probably caused by the Maillard reaction after gelatinization. In this study, the pullulanase used was a brownish liquid, which may also influence the color of ACPB. A similar decrease in color values was observed for oat resistant starch and rice resistant starch [35,52].

### 3.3. Molecular Weight Distribution

The Mw (weight-average molecular weight), Mn (number-average molecular weight) and PDI (polydispersity index) of NB, ACB and ACPB are shown in Table 1. The Mw, Mn and PDI of starch after modification treatment changed remarkably compared to NB. The Mw values of modified starch were in the range of 0.15 × 10^4^~1.90 × 10^4^ KDa, while the Mw value of native starch NB was 4.74 × 10^4^ KDa. The results indicated that the molecular weight of native starch was mainly controlled by amylopectin because of its much larger molecular weight. Additionally, the Mw of ACB was about 1.90 × 10^4^ KDa, which was smaller than that of NB. Meanwhile, the Mw of ACPB was reduced to 0.15 × 10^4^ KDa after combined pullulanase debranching treatment. For all samples, the PDI value for NB was the highest (3.11), followed by ACB (2.82), and ACPB (1.88) was the lowest. Further research should be carried out to evaluate the processing characteristics of black Tartary buckwheat modified starch by amylose chain-length distribution studies.

Significant differences (*p* < 0.05) were found in Mw and PDI for NB and the two modified starches, which may be due to the degradation of starch molecules. During the autoclave modification process, a significant decrease (*p* < 0.05) in the Mw value of ACB appeared, indicating that the starch molecules were degraded under pressure and heat. This may be explained by the mechanism associated with autoclaving that caused a rapid increase in temperature and pressure, which promoted the gelatinization of starch granules, thus leading to the degradation of the long chains of ACB [26]. During the gelatinization of starch, amylose escapes from the starch granules, and the double helical structure is dissociated, thus causing a shift in the molecular weight of the starch [53]. Zhang et al. found that the thermal energy associated with autoclaving led to the degradation of lotus starch molecules, resulting in a significant reduction in the molecular weight of the modified starch [54]. It has been reported that heat-moisture [55], shear and hydrothermal [56] may cause the breakdown of starch molecule and the glycosidic bonds of the starch molecular chains may be disrupted. Compared with NB and ACB, it was found that ACPB displayed the lowest Mw value (0.15 × 10^4^ KDa), suggesting that the debranching treatment had a significant effect on the formation of low Mw in modified starch. Pullulanase could cleave the α-1,6 glucosidic bond of amylopectin, releasing the short chain from the parent amylopectin molecule [57]. Additionally, native starch was a complex molecule with high molecular weight. The PDI reflects the distribution of starch polymers. A larger PDI indicates that the starch is highly dispersed. The lower PDI values of ACB and ACPB suggested that their molecular weight distributions were narrower than that of natural starch NB. In general, a PDI value close to 1.0 indicates a more homogeneous starch component. Comparatively, the high PDI value of NB indicated its broader molecular weight distribution. The combined effect of starch gelatinization and debranching treatment reduced the PDI of native starch. Moreover, the molecular weight dispersion of the debranched modified starch was better. The PDI ranking (NB > ACB > ACPB) was consistent with that of the RS content (discussed in Section 3.8), suggesting that more uniform starch components may be favorable for resistance to enzymatic attack. A similar finding was also reported by Wang et al. [58]. In summary, autoclaving or autoclaving–debranching co-treatment could lead to a reduction in molecular size and more homogeneous molecular weight distribution of native starch.

### 3.4. FTIR

**Figure 3:**
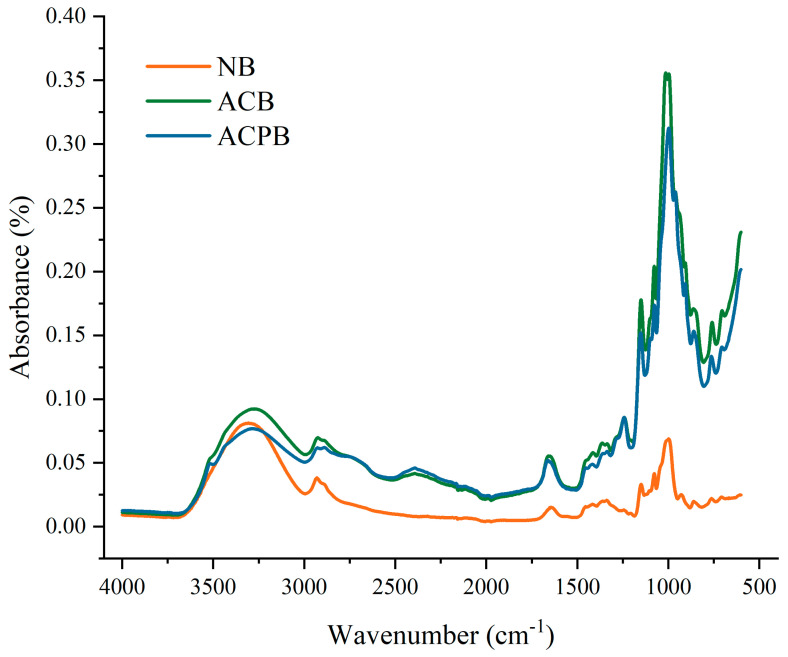
FTIR spectra of NB, ACB and ACPB. NB, black Tartary buckwheat native starch; ACB, black Tartary buckwheat starch subjected to autoclaving treatment; ACPB, black Tartary buckwheat starch subjected to autoclaving–pullulanase combined treatment.

FTIR is sensitive to the molecular structure of starch and serves as a powerful tool for analyzing structural changes during the modification process [51,59]. The short-range ordered structures of autoclaved and autoclaved debranching-modified starches were investigated using FTIR. Compared with NB, the spectra of ACB and ACPB exhibited similar profiles, with no obvious new absorption peaks observed (Figure 3). However, variations in the intensity of certain absorption peaks were evident. The starch samples showed characteristic peaks at 3286 and 2935 cm^−1^, corresponding to -OH and C-H vibrations, respectively. In addition, the spectral region from 800 to 1200 cm^−1^ reflects changes in the short-range ordering of starch. The absorbance ratio at 1047 cm^−1^ to 1022 cm^−1^ (DO) indicates the relative amount of ordered structures in starch, whereas the ratio of absorbance at 995 cm^−1^ to 1022 cm^−1^ (DD) is used to characterize the double helix content in starch. Table 1 summarizes the DO and DD values for NB, ACB and ACPB. The DO value of ACPB increased significantly from 0.7231 to 0.8077 after autoclaving combined with pullulanase treatment compared to single autoclave-treated samples of ACPB. The DD values were recorded as ACPB (1.1807) > NB (1.0935) > ACB (1.0448).

The modified starch and the control sample NB showed similar spectra, which indicated that the chemical bonds and functional groups did not change during the modification process, which only involved the reorientation of the starch chains and the change in intermolecular hydrogen bonding. Similar results were observed in autoclaved arrowroot starch, and the autoclaving process did not alter the chemical structure of the starch [60]. Compared to NB, the intensity of the absorption peak in the 3500–3000 cm^−1^ range for the modified starch increased and broadened. The tensile vibration peaks of hydroxyl groups generally appeared in the range of 3700–3100 cm^−1^ [31]. Therefore, the hydrogen bonding of starch molecules changed significantly during the modification process. In addition, compared to NB, ACPB showed a significant increase in DO values, while the opposite trend was observed in ACB samples. Soler et al. [26] found that autoclave modification treatment led to the disruption of the double helix structure of corn starch, which is consistent with the results of this study. This may also be one of the reasons for the decrease in the DD values of the ACB samples. The experimental results described above support the hypothesis that autoclaving disrupts the ordered structure of starch. The production of more linear chains (Table 1) through autoclaving combined with debranching treatment facilitated the alignment of double-helical structures, resulting in a more ordered arrangement in ACPB-modified starch. Similar results were observed for purple sweet potato starch modified by autoclaving combined with pullulanase [27]. In other words, the combined treatment enhanced the efficient stacking of double-helical structures, which was reflected in an increase in short-range order degree. The above results showed that the combination of autoclaving and pullulanase treatment significantly increased the short-range ordered degree of starch.

### 3.5. WS, SP, WAC and OAC

**Figure 4:**
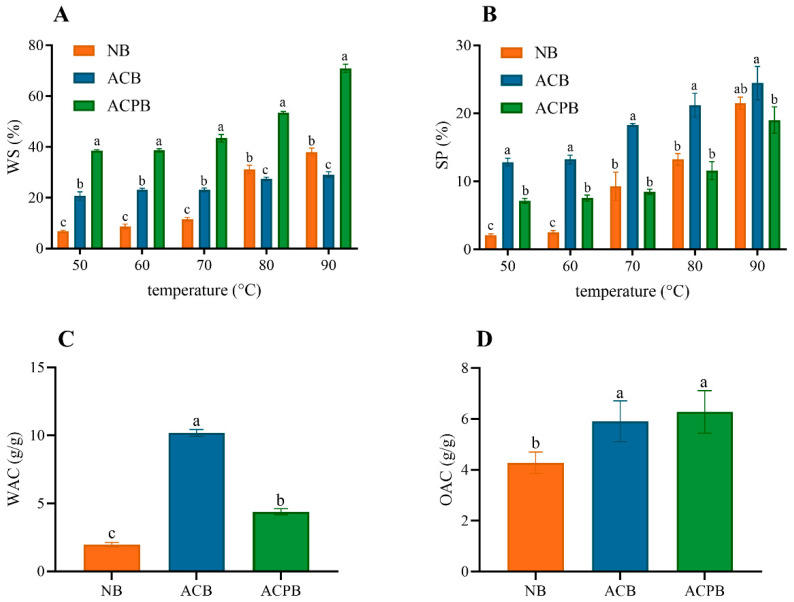
Water solubility (**A**), swelling power (**B**), water absorbing capacity (**C**) and oil absorbing capacity (**D**) of NB, ACB and ACPB. NB, black Tartary buckwheat native starch; ACB, black Tartary buckwheat starch subjected to autoclaving treatment; ACPB, black Tartary buckwheat starch subjected to autoclaving–pullulanase combined treatment. For (**A**,**B**), the means of different letters at the same temperature are significantly different at *p* < 0.05. For (**C**,**D**), the means with different letters are significantly different at *p* < 0.05.

The WS and SP indexes of the native and modified starch samples are shown in Figure 4A,B. Specifically, a general increase in hydration properties with increasing temperature was observed. The WS and SP of all the samples showed an increasing trend as the temperature increased from 50 °C to 90 °C. Compared to the NB and ACPB samples, the WS of the ACB sample was lower (27.40~29.10%) in the range of 80–90 °C. Moreover, the SP of the ACB sample was higher than the other samples in the tested temperature range and was consistent with their particle size variation. The SP of ACB and ACPB was 5.03~22.3% higher than that of NB, with the highest SP value (24.46%) at 90 °C for ACB. The WS of NB ranged from 6.71 to 37.98%, in which ACB and ACPB showed significantly (*p* < 0.05) higher values than NB before 70 °C, whereas ACPB exhibited the highest WS (70.97%) at 90 °C. Results are shown in Figure 4C,D; the highest value of WAC was found for ACB (10.18 g/g), followed by ACPB (4.3 g/g), and NB (11.97 g/g) was the lowest. The OSC of all samples followed the order: ACPB > ACB > NB. Furthermore, the OAC of the NB samples (4.28 g/g) was lower than the modified starch samples (5.91~6.28 g/g).

The WS and SP indexes of NB, ACB and ACPB were measured from 50 to 90 °C at 10 °C intervals. The WS and SP of starch refer to the ability of starch chains to dissolve in water and the degree of water absorption, respectively. WAC and OAC represent the ability of starch molecules to combine with water and oil, respectively. In general, the WS refers mainly to the properties of the released amylose. With the increasing temperature, the starch absorbed water and swelled, the structure dissociated and more linear starch dissolved in water. Therefore, the high WS of ACPB may be due to the increase in linear fragments after pullulanase debranching. Similar results were observed in the debranched arrowhead starch [51]. The breaking of glycosidic bonds and the reduction in intermolecular hydrogen bonds may also cause the increased solubility of starch [61]. It has been reported that SP is related to the fine structure of amylose and amylopectin. Moreover, in terms of SP, the index of all samples increased in the temperature range of 50~90 °C. Xu et al. [62] studied autoclaved chickpea, navy bean and yellow ground pea starches, and they attributed this increase to the starch pasting and dissociation of the crystal structure. ACPB with higher AAC swell less compared to ACB with lower AAC, which is similar to the findings of Hamid et al. [63]. In addition, the WAC of NB increased significantly with increasing temperature after autoclaving and autoclaving–debranching treatments. The WAC is mainly related to the application and modification treatment of starch. The molecular weight results (Section 3.3) showed that autoclaving and autoclaving–debranching resulted in the degradation of the starch chains. Modified starch may be degraded to simple molecules with a high affinity for water, similar to the findings of Marboh and Mahanta [64]. The samples of ACPB showed significantly higher OAC than control NB. This may be due to the entrapment of oil within the helical structure formed by the reorganization in the modified starch [35]. According to Demirkesen-Bicak et al. [65], the increased oil absorption capacity of autoclaved black chickpea starch may be related to the lower protein content of the starch.

### 3.6. Light Transmittance and Freeze–Thaw Stability

**Figure 5:**
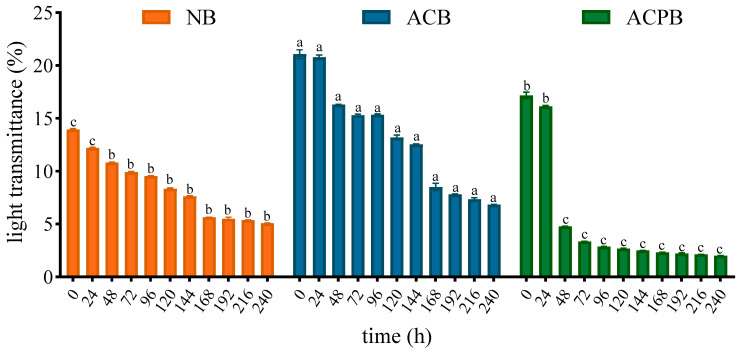
Light transmittance of NB, ACB and ACPB. NB, black Tartary buckwheat native starch; ACB, black Tartary buckwheat starch subjected to autoclaving treatment; ACPB, black Tartary buckwheat starch subjected to autoclaving–pullulanase combined treatment. Data with different letters at the same test time are significantly different at *p* < 0.05.

Figure 5 summarizes the effects of autoclaving and autoclaving–debranching on the transmittance of native starch pastes at low temperatures (4 °C) over a 10-day period. The light transmission of the native and modified starch pastes decreased with increasing storage time, indicating that the turbidity of the starch pastes increased. After the storage period of 10 days, ACPB exhibited the lowest transmittance (2.01%), while the highest transmittance was observed in ACB (6.85%). In this study, the freeze–thaw stability of starch was also investigated. Lower syneresis of starch past represents higher freeze–thaw stability. Syneresis is the amount of water released from the starch paste after the first (1st), third (3rd) and fifth (5th) of the repeated freeze–thawing (FT) cycles. The syneresis value of all samples was significantly increased (*p* < 0.05) during the FT cycles (Table 1). Under three FT cycle treatments, the syneresis value of ACB increased rapidly from 33.10% to 43.06% after the 3rd FT cycle and reached 49.09% after the 5th FT cycle. Meanwhile, ACPB consistently maintained a high syneresis value (57.62~68.26%). The water release rate of all starches increased, and they were ranked in the following order after the 1st, 3rd and 5th FT cycles: ACPB (57.52~68.76%) > ACB (33.10~49.09%) > NB (31.92~40.66%). When the water release at the end of the 1st FT cycle is evaluated, the effect of the autoclaving–debranching treatment was found to be significant (*p* < 0.05).

The light transmission of native and modified starch pastes decreased significantly within 10 days of storage. The changes in the light transmittance could be attributed to the recrystallization of the starch in low-temperature conditions. The time-dependent reduction was intensified by the autoclaving–debranching treatment. This may be explained by the fact that larger numbers of linear chains rearrange more easily than branched starches, leading to more opacities. For the modified starch, the light transmission of ACB was consistently higher than that of the control NB during the equivalent storage, while ACPB showed the opposite trend. Previous studies have shown that the transparency of dextrinized starch was associated with factors such as swelling, distribution of amylopectin chain lengths and intra- and intermolecular bonds [66]. Furthermore, Craig et al. reported that autoclaving caused fragility of the swollen starch granules, which increased the transmittance of starch pastes [67]. Starch with lower water release is more suitable for the frozen food industry. The release of water of native and modified starches increased with the number of FT cycles, which could be attributed to the phase separation that occurred during the freeze–thaw process, resulting in the leakage of water from the gel. However, the modified starches became less resistant to undesirable physical changes during FT cycles. Specifically, the release of water of both ACB and ACPB was significantly higher than that of the control NS and continued to increase during subsequent freeze–thaw cycles. This indicated that the reorganization of molecular chains after autoclaving and autoclaving–debranching treatments did not inhibit the phase separation of starch and water. Therefore, according to the results found in this study, modified starch may not be suitable for frozen food processing.

### 3.7. Rheological Properties of Starch

#### 3.7.1. Steady Shear

**Figure 6:**
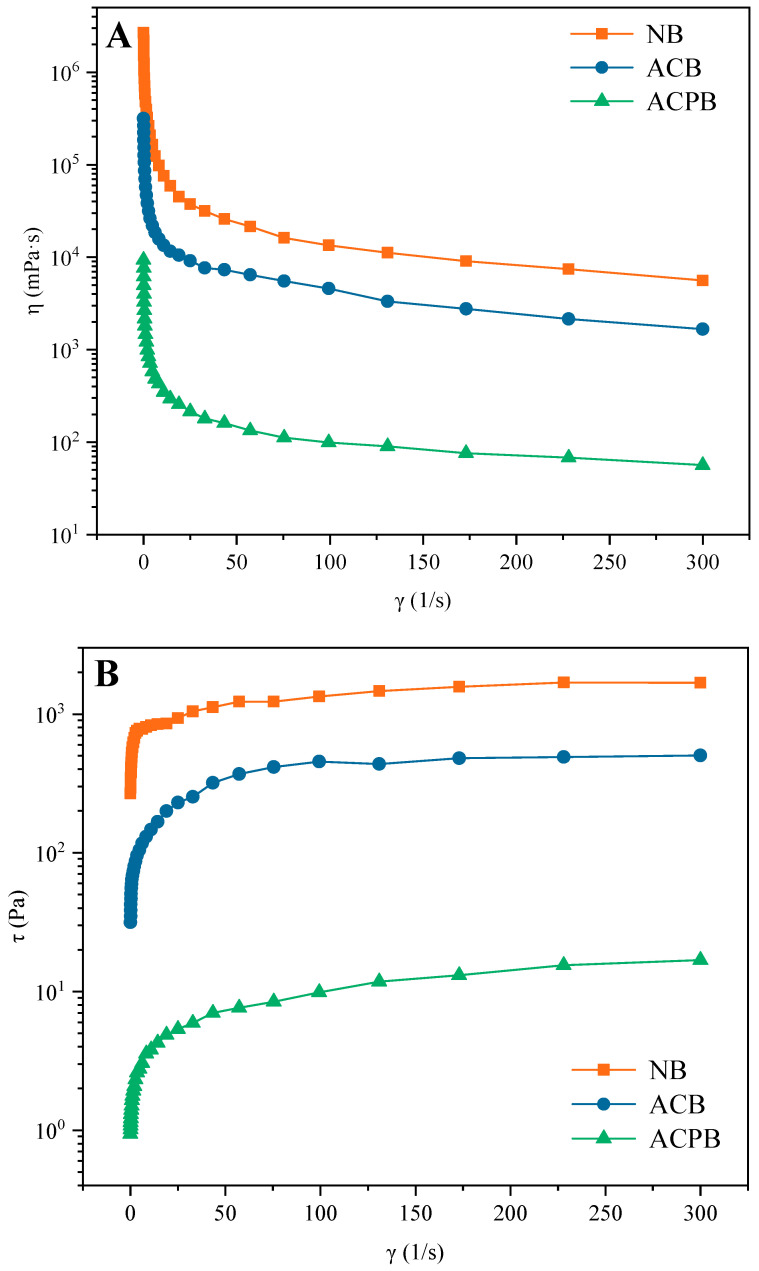
Steady shear flow data. Steady-state shear viscosity (η) (**A**) and shear stress (τ) (**B**) for NB, ACB and ACPB. γ: shear rate; NB, black Tartary buckwheat native starch; ACB, black Tartary buckwheat starch subjected to autoclaving treatment; ACPB, black Tartary buckwheat starch subjected to autoclaving–pullulanase combined treatment.

From the results indicated in Figure 6A, non-linear curves were observed for all starch gels. The apparent viscosities of NB, ACB and ACPB decreased sharply at a low shear rate (0~50 s^−1^) and stabilized at a high shear rate (50~300 s^−1^). The apparent viscosities of ACB and ACPB exhibited lower viscosities when compared with NB. In addition, a further decreased apparent viscosity of ACPB was observed after the debranching treatment. The shear stress–shear rate curves of all starch samples displayed a similar trend. With the increase in shear rate, the shear stress values of NB, ACB and ACPB increased rapidly and showed less fluctuation at larger shear rate values (Figure 6B). Moreover, NB displayed higher shear stress than that of ACB and ACPB. In addition, a power law model was used to fit the steady shear curves of native and modified starches. In this study, the shear thinning behavior of all starches was fitted in a power law model, as shown in Table 1. The rheological data were consistent with the power law model (R^2^ values of 0.968~0.994). Based on the data from the shear stress–shear rate curve fit, it can be seen that the modified starch exhibited lower K values (1.37~71.75) and higher n values (0.36~0.44). The n value of the ACPB product increased (0.44) and exceeded that of the ACB product (0.36) after the pullulanase debranching treatment.

It can be seen that the apparent viscosities of NB, ACB and ACPB decreased with the increase in applied shear stress, thus displaying the phenomenon of shear-thinning, which was the typical characteristic of pseudoplastic fluids. The NB displayed higher shear stress and K values than those of ACB and ACPB, suggesting that its structure needed higher energy to disrupt. Native and modified starches exhibited typical pseudoplastic properties, illustrating that the polymers may disintegrate more rapidly than their reorganization at high shear rates. In addition, the range of mobility coefficient n values indicated that all gels exhibited low viscosity at high shear rates. With further increased shear rates, the starch may be fully oriented and therefore exhibit a stabilized apparent viscosity. In this study, the starch molecular chains were disrupted by heat and pressure during autoclaving (discussed in Section 3.3). Therefore, the reduction in entanglement nodes in the gel may result in a more easily destroyed structure, leading to a decrease in apparent viscosity. Gu et al. suggested that the hydrolysis of starch by pullulanase may lead to a decrease in the interaction with amylose molecules, thereby reducing the apparent viscosity of starch [50]. The flow coefficients (n < 1) also proved their typical shear thinning behavior. Moreover, the decrease in K values could be attributed to the increased number of linear fragments in ACB and ACPB during gelatinization and reorganization. Similar observations were reported in double autoclaved–recrystallized oat starch [52] and heat-treated modified black rice starch [68].

#### 3.7.2. Dynamic Viscoelasticity

**Figure 7:**
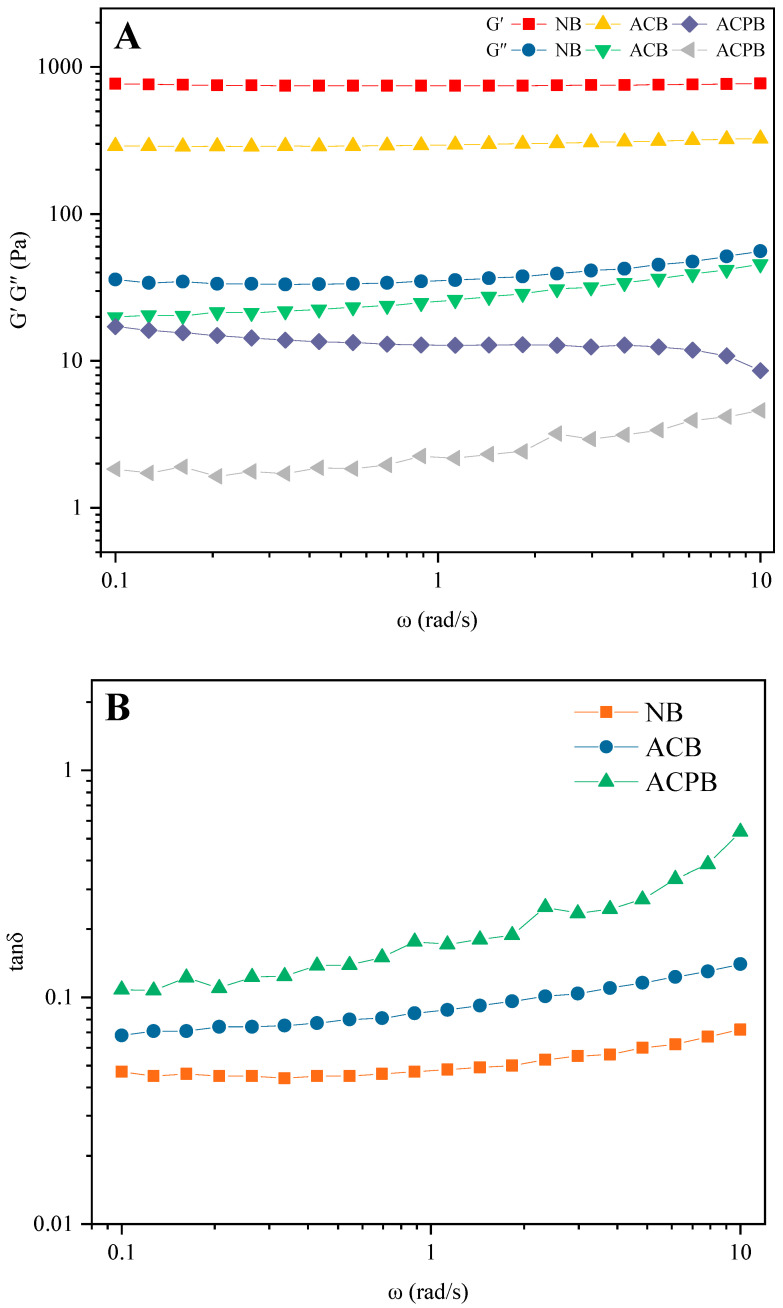
Dynamic frequency sweep data. Storage modulus (G′), loss modulus (G″) (**A**) and loss tangent (tanδ) (**B**) for NB, ACB and ACPB. NB, black Tartary buckwheat native starch; ACB, black Tartary buckwheat starch subjected to autoclaving treatment; ACPB, black Tartary buckwheat starch subjected to autoclaving–pullulanase combined treatment.

The dynamic viscoelasticity of NB, ACB and ACPB gels is shown in Figure 7A; the gel was characterized mainly by the energy storage modulus (G′) and the loss modulus (G″). G′ refers to the elastic state of the system, while G″ represents the viscosity properties of the material. The viscoelastic behavior of the starch was determined at different angular frequencies. All samples displayed gel-like viscoelastic behavior (G′ > G″). The G′ values of both modified starches were lower than those of native starch gels, indicating that ACB and ACPB modified starches were less rigid than NB. Moreover, the modified starches showed lower G′ and G″ than that of the control group. In addition, the G′ value of ACB was lower than that of native starch gel after autoclaving treatment, and after combined debranching treatment, the G′ of ACPB was drastically reduced. This also suggests that the magnitude of the elastic properties of modified starch depends on the changes in the starch chains. The tanδ can be assessed for the elasticity and viscosity of the gel. The tanδ of each modified starch was less than 1 under the tested conditions (Figure 7B) and showed elastic behavior. The tanδ of NB gels was increased after autoclaving or combined debranching modification treatments. The tanδ of ACB gels was significantly higher than that of NB and lower than that of ACPB gels. In addition, the tanδ value of ACPB was higher than that of ACB and NB, indicating that the elasticity of the starch gel was reduced after the debranching treatment.

Compared to the NB gels, the G′ and G″ of the ACB gels decreased, and further decreases were observed for the ACPB gels. This suggested that both modification treatments reduced the viscoelasticity of the gels, especially the debranching effect of pullulanase. This effect may be due to the lowered starch granule integrity (Figure 1(B2,C2)). Similar results were found by Geng et al. [69]. By increasing the amount of pullulanase, the G′ and G″ of rice starch co-modified by preheating and pullulanase were further reduced. After retrodegradation, the reorganized starch chains might not be fully unfolded during re-gelatinization, resulting in ACB and ACPB having lower elastic properties [30,70,71]. Moreover, it was observed that G′ was higher than G″ in all starch gels, which was consistent with the results reported by Navaf et al. [72]. They found that the modified *Corypha umbraculifera* L. starch subjected to different hydrothermal treatments exhibited the typical weak gelation behavior. At angular frequencies less than 1, all samples exhibited a smaller tanδ, reflecting their elastic behavior. By increasing the measurement frequency, all samples became more liquid-like. However, the tanδ of the modified starch was higher than that of the native starch, indicating that both autoclaving and autoclaving–debranching treatments reduced the elasticity of the gels. In particular, the debranching of pullulanase formed more linear chains, and their interactions further weakened the gel network structure. The strength of the hydrogel decreased with the production of linear short amylose due to differences in molecular entanglement [73]. This may explain why the tanδ of ACB is smaller than that of ACPB. A similar result was observed by Geng et al. [69], who investigated the rheological properties of rice starch modified by preheating and pullulanase treatments.

### 3.8. In Vitro Digestibility

**Figure 8:**
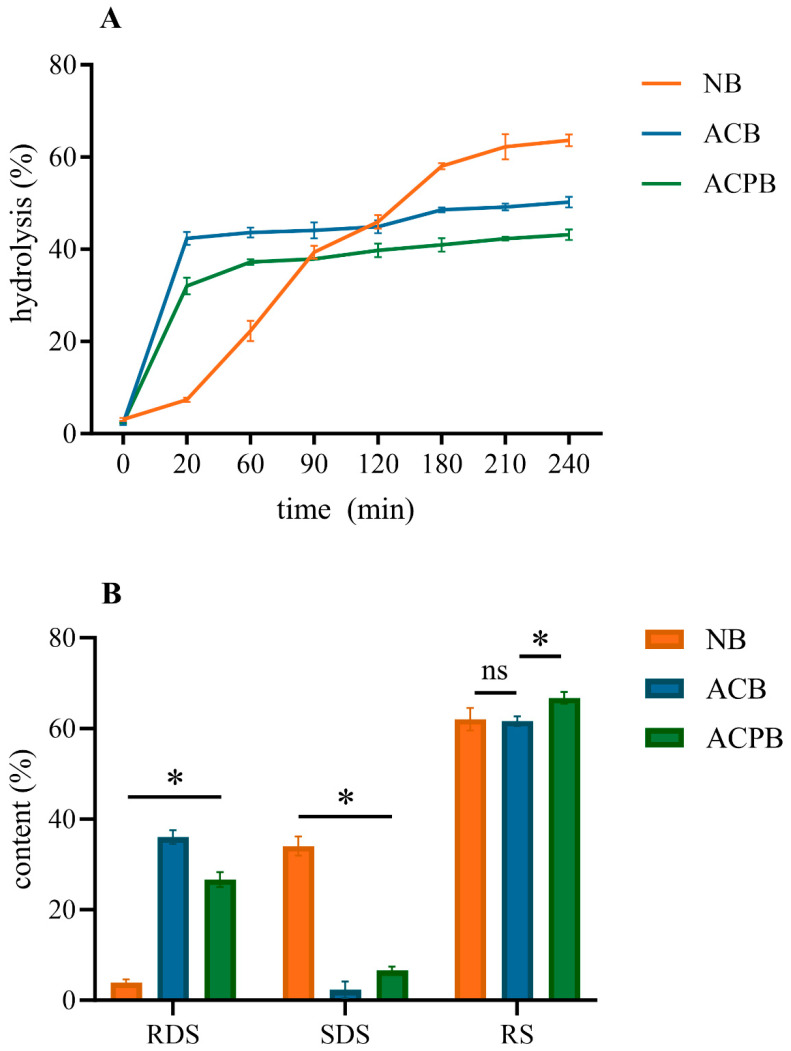
Digestibility of NB, ACB and ACPB. Hydrolysis curve (**A**); rapidly digestible starch (RDS), slowly digestible starch (SDS), and resistant starch (RS) of samples (**B**). NB, black Tartary buckwheat native starch; ACB, black Tartary buckwheat starch subjected to autoclaving treatment; ACPB, black Tartary buckwheat starch subjected to autoclaving–pullulanase combined treatment. ns: the means are not significantly different at *p* > 0.05 with each other. *: the means are significantly different at *p* < 0.05 with each other.

**Figure 9:**
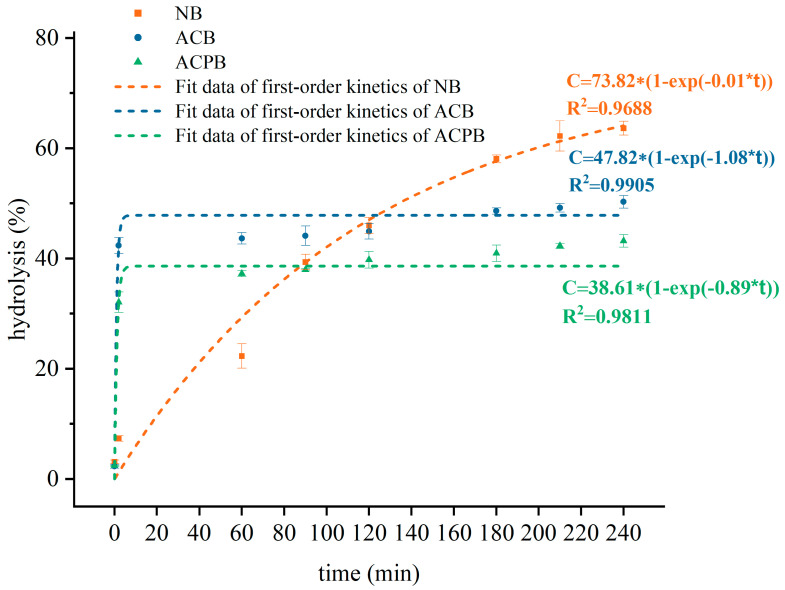
Fit data of first-order kinetics of NB, ACB and ACPB. NB, black Tartary buckwheat native starch; ACB, black Tartary buckwheat starch subjected to autoclaving treatment; ACPB, black Tartary buckwheat starch subjected to autoclaving–pullulanase combined treatment. C (%) is the total amount of starch digested at digestion time t; C∞(%) is the total amount of starch digested at 240 min.

The in vitro digestibility of NB, ACB and ACPB is presented in Figure 8A. The digestive hydrolysis rates of all samples changed significantly in the first 20 min, and the digestion rates of NB, ACB and ACPB at 20 min were 7.34%, 42.35% and 32.05%, respectively. The final hydrolysis rates of NB, ACB and ACPB were recorded as follows: NB (63.62%) > ACB (50.27%) > ACPB (43.18%). In addition, the hydrolysis rates of modified starches tended to be equilibrated after 180 min. The results of fitting data with first-order kinetics for all samples are shown in Figure 9. All starches displayed a satisfactory fit, with R^2^ values from 0.968 to 0.994. K represents the hydrolysis rate constant. According to the fitting equation: C = C∞(1 − e^kt), the K values for ACB and ACPB were 1.08 and 0.89, respectively, indicating that the debranching treatment reduced the hydrolysis rate of the ACPB samples. The RDS, SDS and RS contents of native and modified starches are shown in Figure 8B. The components of the modified starches showed significant differences compared to native starches. The RDS, SDS and RS contents of NB were 3.87%, 34.05% and 62.08%, respectively. However, the RDS of the modified starch was 22.81~32.18% higher than that of the native starch. The SDS of all samples followed the order: NB (34.05%) > ACPB (6.55%) > ACB (2.33%). In addition, ACPB showed the highest RS content (66.77%). No significant differences were observed in the RS content of native starch and ACB (*p* > 0.05).

There was a significant difference in the RDS content of native and modified starches, which suggested that the variations in the structure of starch may affect their availability to digestive enzymes. Previous studies have shown that starch digestibility is strongly influenced by its structural integrity [74]. The destruction of native starch granules resulted in a significant increase in RDS content. A similar change was previously reported on purple sweet potato starch subject pullulanase debranching combined with autoclaving treatment [75]. Moreover, the modified starches exhibited lower hydrolysis rates in the entire simulated digestion process when compared to NB, indicating that the internal structure of the modified starch became more resistant to enzymatic hydrolysis. Therefore, the increased RS content in modified starch may play a dominant role in starch resistance to digestion compared to that of the RDS. In addition, the lower digestion rate of ACPB compared to that of ACB may be attributed to the following: (1) linear chains can move quickly and then aggregate themselves to form more compact structures [27]; (2) a narrowing of the molecular weight distribution may promote the crystallization of starch, thus leading to a stronger resistance of recrystallized starch [76]; (3) relatively ordered structures (e.g., the significantly increased DO of ACPB) may be more resistant to enzymatic attack [77]. Similar results have been reported by Yang et al. [78], who found that the hydrolysis rate of euryale ferox resistant starch prepared via enzymatic autoclaving was lower compared to that prepared by autoclaving alone. Therefore, the variation in molecular weight had a significant effect on the digestibility of modified starch. Moreover, modified starches may help to maintain low digestibility over a longer period of time.

## 4. Conclusions

The changes in the morphology, particle size, hydration properties, rheological properties and digestibility of black Tartary buckwheat starch modified by autoclaving combined with pullulanase have been revealed. This study indicated that the autoclaving combination with pullulanase treatment may improve the processing properties of black Tartary buckwheat starch and enhance its digestibility resistance. Significant differences were observed in the molecular weight for NB and the two modified starches. These differences induced their different physicochemical structures, such as irregular structure with a large size, weaker elasticity, reduced viscosity, increased solubility and varying degrees of changes in solubility, swelling power and water- and oil-absorption capacities. Moreover, ACPB had the lowest digestibility and highest DO value. Autoclaving combined with pullulanase led to an increase in AAC and a further decrease in Mw. During retrodegradation, the narrower molecular weight distribution and more linear chains may contribute to the increased mobility of molecular chains, promoting the reorganization and recrystallization process and resulting in a higher resistance of modified starch against enzymatic digestion. The research results obtained in this study may provide some reference for the application of black Tartary buckwheat modified starch in industry. Further studies could investigate the application of ACB and ACPB with diverse physicochemical properties in various food processes, including the production of snacks, beverages and fried puff pastry.

## Figures and Tables

**Table 1:** Apparent amylose content**,** molecular weight, molecular order, particle size distribution, parameters of amylose distribution, color, freeze–thaw stability and parameters of power law of NB, ACB and ACPB. NB, black Tartary buckwheat native starch; ACB, black Tartary buckwheat starch subjected to autoclaving treatment; ACPB, black Tartary buckwheat starch subjected to autoclaving–pullulanase combined treatment. D[4,3]: the average diameter of the area; D[3,2]: the average diameter of the volume; Mw: weight-average molecular weight; Mn: number-average molecular weight; Mp: peak molecular weight; PDI: polydispersity index; DO: the ratio of absorbance of the sample at 1047 and 1022 cm^−1^; DD: the ratio of absorbance of the sample at 995 and 1022 cm^−1^. L*: lightness, a*: redness, b*: yellowness; 1st: first repeated freeze–thawing cycle; 3rd: third repeated freeze–thawing cycle; 5th: fifth repeated freeze–thawing cycle; K: consistency factor; n: mobility behavior index; R^2^: fitting coefficients under the power law model. Means in the same row with different letters are significantly different at *p* < 0.05.

Parameter	Samples		
	NB	ACB	ACPB
Apparent amylose content (%)	12.95 ± 0.36 c	16.28 ± 0.75 b	24.15 ± 0.72 a
Size distribution (μm)			
peak particle size	9.57 ± 0.12 c	155.60 ± 1.01 a	105.53 ± 0.23 b
D[4,3]	15.12 ± 0.43 c	136.13 ± 0.38 a	103.37 ± 0.32 b
D[3,2]	5.36 ± 0.02 c	66.73 ± 0.59 a	47.12 ± 0.23 b
Molecular weight			
Mw (×10^4^ KDa)	4.74 ± 0.10 a	1.90 ± 0.16 b	0.15 ± 0.00 c
Mn (×10^4^ KDa)	1.52 ± 0.09 a	0.67 ± 0.04 b	0.08 ± 0.00 c
Mp (×10^4^ KDa)	17.40 ± 0.54 a	0.68 ± 0.04 b	0.07 ± 0.00 b
PDI	3.11 ± 0.14 a	2.82 ± 0.06 b	1.88 ± 0.03 c
Molecular order			
DO	0.7276 ± 0.0146 b	0.7231 ± 0.0211 b	0.8077 ± 0.0163 a
DD	1.0935 ± 0.0188 b	1.0448 ± 0.0150 c	1.1807 ± 0.0095 a
Color attributes			
L*	98.99 ± 0.46 a	97.01 ± 0.58 c	97.66 ± 0.15 b
a*	−0.74 ± 0.07 b	−0.56 ± 0.10 a	−0.54 ± 0.07 a
b*	2.42 ± 0.13 c	2.71 ± 0.10 b	3.43 ± 0.07 a
Freeze–thaw stability (%)			
1st cycles	31.92 ± 0.93 b	33.10 ± 1.49 b	57.62 ± 2.16 a
3rd cycles	34.19 ± 1.09 c	43.06 ± 1.45 b	63.71 ± 0.81 a
5th cycles	40.66 ± 2.13 c	49.09 ± 2.21 b	68.26 ± 3.55 a
Power law model			
K (Pa·s^n^)	515.99 ± 11.63 a	71.75 ± 5.60 b	1.37 ± 0.06 c
n	0.21 ± 0.01	0.36 ± 0.02	0.44 ± 0.01
R^2^	0.986	0.968	0.994

## Data Availability

The original contributions presented in the study are included in the article, further inquiries can be directed to the corresponding author.
